# Expanding the “Magic Triangle” of Reinforced Rubber Using a Supramolecular Filler Strategy

**DOI:** 10.3390/ma16093429

**Published:** 2023-04-27

**Authors:** Yihong Zhao, Mingwei Ren, Xiangdong Zhu, Zhangyu Ren, Yaofang Hu, Huhu Zhao, Weiheng Wang, Yunbo Chen, Kewei Gao, Yujing Zhou

**Affiliations:** 1 State Key Laboratory of Advanced Forming Technology and Equipment, China Academy of Machinery Science and Technology Group, Beijing 100083, China; 2 Dezhou Branch of Beijing National Innovation Institute of Lightweight Ltd., Dezhou 253049, China; 3 School of Materials Science and Engineering, University of Science and Technology Beijing, Beijing 100083, China

**Keywords:** supramolecular filler, magic triangle, β-alanine, rubber composites

## Abstract

A strategy for optimizing the rolling resistance, wet skid and cut resistance of reinforced rubber simultaneously using a supramolecular filler is demonstrated. A β-alanine trimer-grafted Styrene Butadiene Rubber (A_3_-SBR) pristine polymer was designed and mechanically mixed with commercially available styrene butadiene rubber to help the dispersion of a β-alanine trimer (A_3_) supramolecular filler in the rubber matrix. To increase the miscibility of A_3_-SBR with other rubber components during mechanical mixing, the pristine polymer was saturated with ethanol before mixing. The mixture was vulcanized using a conventional rubber processing method. The morphology of the assembles of the A_3_ supramolecular filler in the rubber matrix was studied by Differential Scanning Calorimetry (DSC) and Transmission Electron Microscopy (TEM). The Differential Scanning Calorimetry study showed that the melting temperature of β-sheet crystals in the vulcanizates was around 179 °C and was broad. The melting temperature was similar to that of the pristine polymer, and the broad melting peak likely suggests that the size of the crystals is not uniform. The Transmission Electron Microscopy study revealed that after mixing the pristine polymer with SBR, some β-sheet crystals were rod-like with several tens of nanometers and some β-sheet crystals were particulate with low aspect ratios. Tensile testing with pre-cut specimens showed that the vulcanizate containing A_3_-SBR was more cut-resistant than the one that did not contain A_3_-SBR, especially at a large cut size. The rolling resistance and wet skid were predicted by dynamic mechanical analysis (DMA). DMA tests showed that the vulcanizates containing A_3_-SBR were significantly less hysteretic at 60 °C and more hysteretic at 0 °C based on loss factor. Overall, the “magic triangle” was expanded by optimizing the rolling resistance, wet-skid, and cut resistance simultaneously using a β-alanine trimer supramolecular filler. The Payne effect also became less severe after introducing the β-alanine trimer supramolecular filler into the system.

## 1. Introduction

**Scheme 1:**
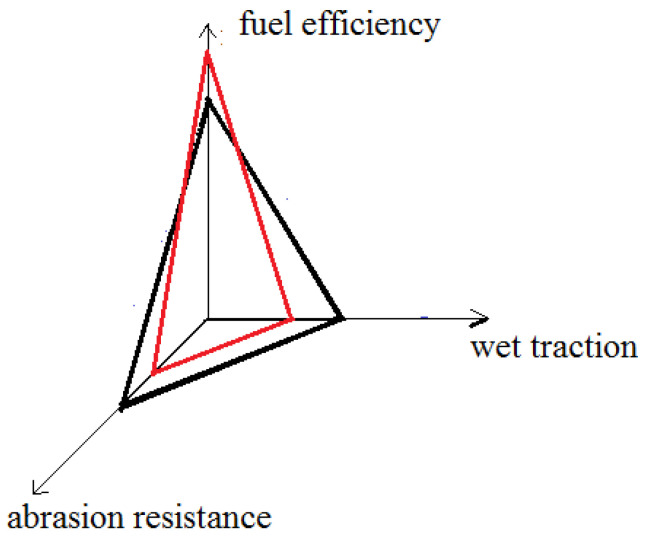
Schematic illustration of the “magic triangle” problem.

Elastomers are inherently soft and weak and must be reinforced for practical applications [1]. The reinforcement of elastomers is usually achieved by introducing rigid entities of a sub-micrometer scale or nanometer scale into elastomers [2,3,4,5,6,7,8,9,10]. In vulcanized elastomers, the most common fillers are carbon blacks and silica, which are pre-formed particles and are dispersed by mechanical mixing [11,12,13]. However, for reinforced vulcanizates reinforced by the common fillers, there is always a “magic triangle” problem (Scheme 1), and the rolling resistance, wet traction and abrasion resistance can be optimized only under mutual impairment [14,15,16]. The improvement of one characteristic means impairing another.

Oligo(β-alanine)s are potentially an ideal filler for solving the “magic triangle” problem. They have an extraordinary propensity for forming β-sheets via intermolecular hydrogen bonds. The β-sheets stack to form crystals via van der Waals force and dipole–dipole interaction [17,18]. When the β-alanine segments are covalently bonded with rubber chains, microphase separation occurs to give β-sheet nanocrystals dispersed in a continuous rubber phase [19]. The non-covalent interactions in the supramolecular fillers are well-defined and tunable in contrast to the traditional fillers such as carbon black and silica. The filler–filler and filler–polymer interactions for carbon black and silica are ill-defined, resulting in an unnecessary waste of hysteretic energy. In the previous study of the Jia group [20,21,22,23], oligo(β-alanine)s were used as hard segments in thermoplastic elastomers. They proved that the oligo(β-alanine)s formed nano-sized supramolecular hard domains and improved tensile strength and reduced rolling resistance significantly.

**Scheme 2:**
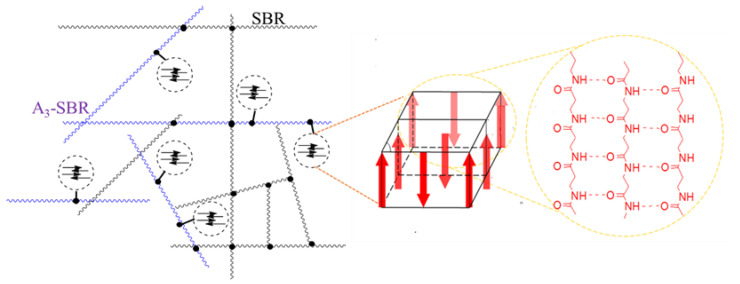
A scheme shows the supramolecular filler dispersed in the rubber matrix. Each arrow represents for a strand of oligo(β-alanine)s molecule.

In this study, we focused on expanding the “magic triangle” by using a supramolecular filler strategy. This new strategy involved replacing part of conventional filler with oligo(β-alanine)s supramolecular fillers in rubber vulcanizates. The oligo(β-alanine)s have high polarity, and they intend to form micro-sized aggregates which act as defects in the rubber matrix. Therefore, oligo(β-alanine)s must be covalently connected to rubber chains to obtain good dispersion. Therefore, we synthesized the β-alanine trimer-grafted SBR (A_3_-SBR) pristine polymer, and mixed the pristine polymer with commercially available styrene butadiene rubber to help dispersion (Scheme 2). At a relatively low amplitude of strain and frequency, the oligo(β-alanine) supramolecular fillers were expected to form β-sheet nanocrystals and the crystals were expected to compensate for the breakage of the filler–filler interaction of the carbon black and, as a result, delay the reduction of the storage modulus and reduce the Payne effect. Furthermore, the nanocrystals were covalently bonded to the soft phase. This would minimize the mechanical energy loss and, hence, keep rolling resistance low. At a high amplitude of strain and frequency, the crystals should undergo deformation and absorb mechanical energy. This was expected to enhance wet-skid and retard the crack growth.

## 2. Materials and Methods

### 2.1. Materials

All solvents and reagents were purchased from Oakwood (Estill, SC, USA) or Sigma-Aldrich (St. Louis, MO, USA). All except triethylamine were used as received. Triethylamine was dried over Na/K for 24 h and distilled prior to use under nitrogen protection. Styrene-butadiene copolymer (SLF16S42) and polybutadiene (PBD) were donated by Goodyear Tire & Rubber Company (Akron, OH, USA). Stearic acid, zinc oxide, sulfur, retarder, wax, antioxidant and accelerator were donated by Akrochem (Akron, OH, USA). Carbon black N115 (STSA surface area 123 m^2^/g, iodine adsorption number 160 mg/g) was provided by Cabot Corporation. All reagents and solvents for synthesis were purchased from Sigma-Aldrich and were used as received.

### 2.2. Synthesis of Pristine Polymer

**Scheme 3:**
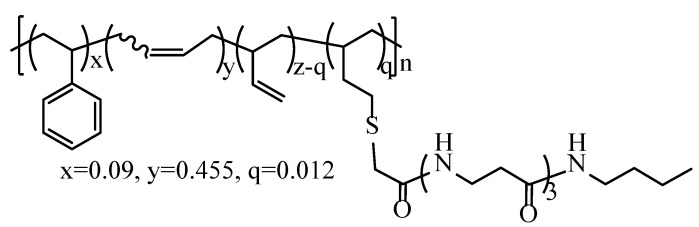
Structure of pristine polymer A_3_-SBR.

The synthesis route of pristine polymer A_3_-SBR (Scheme 3) was reported in the previous work. A solution polymerized SLF16S42 styrene butadiene copolymer was chosen as polymer backbone because of the following 3 reasons. First, SLF16S42 has high vinyl content (42 wt %) for thiol-ene reaction. Second, SLF16S42 has no oil content. Third, SLF16S42 has no gel content. The synthesis steps were the same as those of the polymer **3a** in the previous work [20] and produced a β-alanine trimer loading of 5.5 wt % calculated based on elemental analysis result. The β-alanine trimer loading was slightly different to the polymer **3a** in the previous work because of the batch-to-batch variation.

### 2.3. Compounding and Vulcanization of βA20–CB40, Rf and βA20 Composites

A_3_-SBR saturated with ethanol was used for mechanical mixing. The ethanol was expected to act as a plasticizer for the β-alanine component and increase the miscibility of A_3_-SBR with other rubber components. The ethanol-saturated A_3_-SBR was prepared by precipitating a chloroform–ethanol solution (volume ratio = 10:1) A_3_-SBR in ethanol. The ethanol on the surface of the polymer was wiped with a paper towel. The weight ratio of A_3_-SBR to ethanol was 8:6 in the resulting precipitate.

The formulations of rubber compounds are summarized in Table 1. Rf did not contain A_3_-SBR and was used as a reference sample to match the stress–strain curve of βA20–CB40. βA20 did not contain carbon black and was used as a sample for TEM study. An 80 cc Brabender mixer was used to mechanically mix SBR composites. Fill factor was 0.7–0.8. Rotor speed was 80 rpm. Initial temperature was 60 °C, and dump-out temperature was about 95 °C. Mixing procedure was as follows: 0–1.5 min stryrene butadiene copolymer, PBD and A_3_-SBR, 1.5–4 min ½ carbon black N115, 4–12 min zinc oxide, stearic acid, antioxidant DQ, retarder CTP, antiozonant PD-2, ½ carbon black, 12–15 min wax. The compound was dumped at 95 °C.

After mixing, sulfur and BBTS were added to the compound on a two roll mill. Roll speeds were 8 rpm for slow roll and 12 rpm for the fast roll. Compounds were milled at 60 °C for one minute and then curatives were added to the bank with alternating cuts on both sides. After 20 end roll passes, milled sheets were taken off.

**Figure 1:**
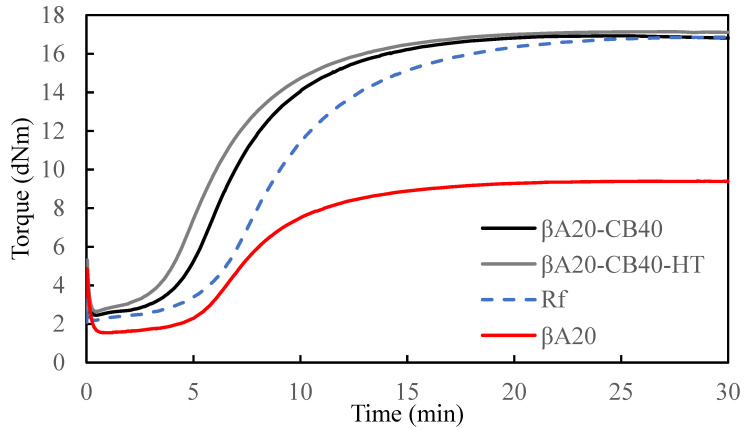
Curing curves of βA20–CB40, βA20–CB40–HT, βA20 and Reference sample (Rf).

The milled sheets were relaxed overnight at room temperature. About 5 g of the sample was cut from the milled sheet and taken for a Moving Die Rheometer (MDR) test at 160 °C to obtain vulcanization kinetics. (Figure 1). The curing parameters are summarized in Table 2. The specimens for mechanical properties tests were compression molded. The milled sheets were cured at 160 °C for [t(90) + 10] min in a Dake hydraulic press under a load of 35 tons and then quenched in water.

### 2.4. Compounding and Vulcanization of βA20–CB40–HT Composite

βA20–CB40–HT was compounded to show the effect of mixing methods on material properties. The formulation for βA20–CB40–HT was the same as the βA20–CB40, and the mixing procedures for the two were different. βA20–CB40–HT was compounded following the common compounding strategy for styrene butadiene rubber. The pristine polymer was directly mixed with the other rubber components without ethanol saturation. The initial temperature was 90 °C, and the rotor speed was adjusted during mixing to obtain a dump-out temperature of around 160 °C.

### 2.5. Characterization Methods

The β-alanine trimer content of A_3_-SBR pristine polymer was calculated based on elemental analysis result, and the test was carried out by Micro-Analysis INC., Wilmington, DE, USA. The sample was combusted in pure oxygen; the gases were carried through the system by helium, converted and measured as CO_2_, H_2_O, N_2_ and SO_2_. The gases were separated under steady-state conditions and were detected by Thermal Conductivity or IR. The weight percentage of different elements in the A_3_-SBR pristine polymer are given in Table 3.

The weight percentage of β-alanine trimer in the A_3_-SBR pristine polymer was calculated using the following equation:

(1) $$\frac{256\times N\%}{56}\;$$

The weight percentage of β alanine trimer in the vulcanized mixture was then calculated using the following equation:

(2) $$\frac{50\times A_{3}\%}{151.4}\;$$

Phase separation of β-alanine trimer was characterized using DSC. The experiment was carried out on a TA instrument model Q2000 (New Castle, DE, USA) under nitrogen. About 5 to 8 mg of each sample was placed in a T-zero aluminum pan. The sample was heated from −80 °C to 250 °C under nitrogen with heating rate at 10 °C/min.

Morphology was studied using a transmission electron microscope (FEI Tecnai F20 field emission instrument, 200 kV, FEI company, Hillsboro, OR, USA). The compression-molded vulcanizate was microtomed at −70 °C using an ultracryomicrotome (Leica EM UC7, Leica Microsystems Inc., Deerfield, IL, USA) to obtain slices with around 80 nm thickness. Continuous ultrathin carbon film coated lacey carbon supported copper grids were used to hold the thin slices. Only areas supported by the ultrathin carbon film (5–10 nm thick) were observed in order to reduce background. A modified low-dose mode was employed in order to minimize the radiation damage during imaging [24].

Tensile properties of SBR composites were tested using an Instron model 5567 (Norwood, MA, USA). Tensile specimens were cut with an ASTM D 412-89 Type C dumbbell die. Three tensile specimens were tested for each case. The crosshead speed was 50 mm/min with an initial grip separation of 65 mm. Stress was calculated based on the initial specimen width and thickness. A clip-on extensometer was used to measure displacement. Strain was calculated based on the displacement and the initial distance between two clips. Stress–strain curves were recorded.

The cut resistance study was carried out using pre-cut samples. An edge-cut was made with a sharp razor blade midway along each specimen. Cut sizes were 0.1–3 mm. These specimens were subjected to tensile extension. Test conditions were the same as those used in normal tensile tests.

Rolling resistance and wet-skid resistance were predicted using dynamic mechanical analysis with temperature sweep mode. Tanδ at 0 °C and 60 °C are commonly taken as indicators for wet skid resistance [25] and rolling resistance [26,27] of tire tread. The experiment was performed on DMA (TA Q800) in tension mode in the temperature range of −60 °C to 200 °C. The heating rate was 10 °C/min. Samples were tested under 1 Hz frequency and 2% oscillatory strain. The storage modulus (E′), loss modulus (E″) and loss factor (tan δ) were recorded.

The Payne effect was analyzed by dynamic mechanical analysis with strain sweep mode. The tests were carried out at a frequency of 1 Hz at room temperature (25 °C). The strain amplitude was from 0.01% to 10%.

The crosslinking density of βA20–CB40 and βA20–CB40HT was determined by swelling test. Five pieces of cured sheet of βA20–CB40 and βA20–CB40HT (about 0.2 g for each piece) were swelled with toluene in the dark at room temperature for one week. Swollen pieces were then wiped with a paper towel and immediately weighed. Then, the pieces were weighed again after drying in a vacuum oven at 50 °C overnight. Crosslink density was calculated using the Flory–Rehner equation [28]:

$$\rho =-\frac{1}{2\nu s}\times \frac{\ln(1-\nu_{r}^{0})+\nu_{r}^{0}+\chi ({\nu_{r}^{0})}^{2}}{\sqrt[{3}]{\nu_{r}^{0}}-\frac{1}{2}\nu_{r}^{0}},$$

where ρ is the crosslink density, ν_s_ is the molar volume of the swelling solvent toluene (1.07 × 10^−4^ m^3^/mol at 25 °C) and χ is the interaction parameter for toluene/rubber (0.32 for filled SBR). ν_r_^0^ is the volume fraction of unfilled rubber in the swollen gel. ν_r_^0^ can be calculated from the following equation [29]:

$$\frac{\nu_{r}^{0}}{\nu_{r}}=1-\left[3c\left(1-\sqrt[{3}]{\nu_{r}^{0}}\right)+\nu_{r}^{0}\;-1]\phi /(1-\phi \right),$$

where ν_r_ is the volume fraction of filled rubber in the swollen rubber phase and c is a parameter depending on the filler (c = 0.127 for super abrasion furnace carbon black; the value was calculated based on a swelling test of filled vulcanizates by G. Kruaus [29]. c has some dependence on the carbon black aggregate structure). ϕ is the volume fraction of filler. ϕ can be calculated from the weight and density of ingredients using the following equation:

ϕ = (m_CB_ ÷ d_CB_)/(m_1_ ÷ d_1_ + …… + m_n_ ÷ d_n_),

where m_CB_ is the weight of filler, d_CB_ is the density of filler (1.8 g/cm^3^ for carbon blacks), m_n_ is the weight of each ingredient and d_n_ is the density of each ingredient. ν_r_ can be calculated from the swollen weight and dried weight using the following equation:

ν_r_ =V_R_/(V_R_ + V_S_),

where Vs = (W_gel_ − W_dry_)/d_toluene_, V_filler_ = W_initial_(F_CB_/d_CB_), V_R_ = (W_dry_/d_dry_) − V_filler_, V_R_ is the polymer volume, Vs is the solvent volume, d_toluene_ is the density of toluene (0.862 g/cm^3^), d_CB_ is the density of carbon black (1.8 g/cm^3^) and F_CB_ is the weight fraction of carbon black in the formulations.

## 3. Results

### 3.1. Phase Separation of Filler in SBR Composite

**Figure 2:**
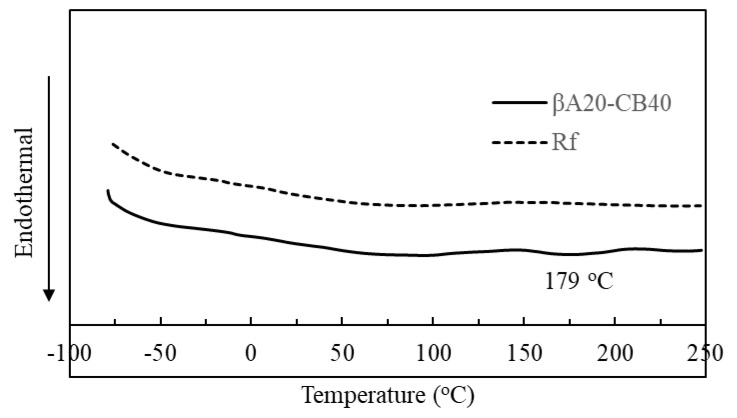
DSC curve of βA20–CB40 and Rf.

We have previously reported the microphase separation of A_3_ in the pristine polymer [24], and the melting temperature for the pristine polymer was ~183 °C. The DSC curve of βA20–CB40 and reference sample (RF) was compared in Figure 2. βA20–CB40 showed a broad endotherm peak at around 179 °C, while Rf did not show a peak at ~179 °C, indicating that A_3_ was crystallized and phase separated from the rubber matrix in βA20–CB40. The endotherm peak at around 179 °C was attributed to the melting of the crystalline β-sheet microphase in βA20–CB40, and coincided with the melting peak of the β-sheet crystal in the pristine polymer. The melting peak at around 179 °C was broad, indicating that the crystal size was not unified. The broad peak below 120 °C for both βA20–CB40 and Rf corresponded to the melting of stearic acid and wax.

### 3.2. Morphology of Filler in the SBR Composite

**Figure 3:**
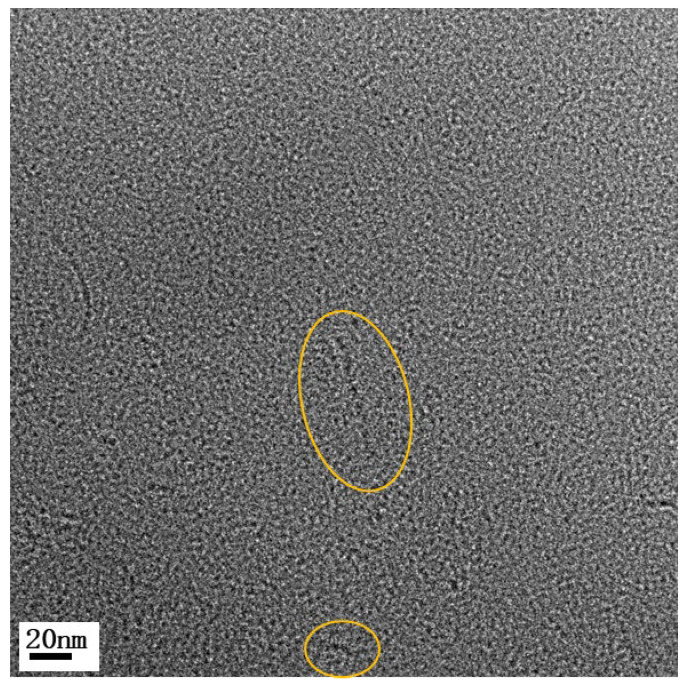
TEM image of βA20. Features in circle show rod-like and particulate crystals.

Vulcanized βA20 was microtomed at −70 °C to ~80 nm thin slices and studied using TEM. βA20 contains the same ingredients as βA20–CB40, except that βA20 does not contain carbon black, so the A_3_ crystal morphology is able to be observed. As shown in Figure 3, after mixing the pristine polymer with SBR and vulcanizing, A_3_ still formed rod-like crystal. The rods were a few nanometers wide and a few tens of nanometers long. Particulate features also existed, coinciding with the broad melting peak at around 179 °C, shown in the DSC curve.

### 3.3. Tensile Properties and Cut Resistance Study

**Figure 4:**
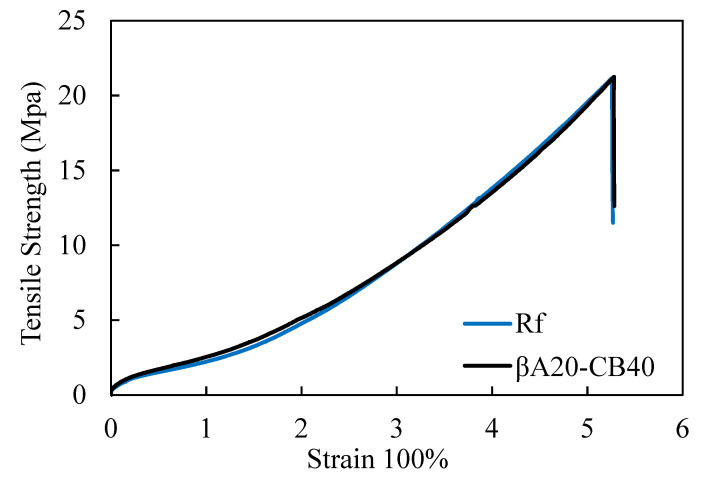
Stress–strain curve of βA20–CB40 and reference sample.

The carbon black content of the reference sample was the same as the βA20–CB40, while the reference sample did not contain A_3_-SBR. The curing package of the reference sample was adjusted to match the tensile properties of βA20–CB40, so that we could compare the other properties of βA20–CB40 with the reference sample. The stress strain curves of βA20–CB40 and reference sample “Rf” are shown in Figure 4. The tensile properties are summarized in Table 4. The strength at 300% strain, the ultimate strain and the ultimate strength of βA20–CB40 matched well with the reference sample.

**Figure 5:**
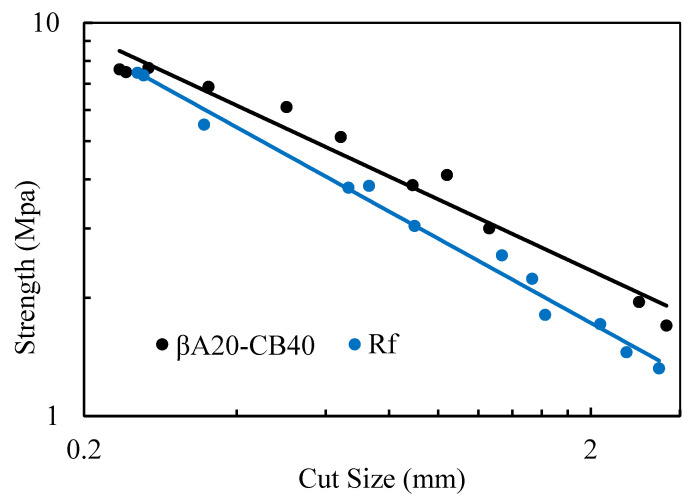
Cut resistance of βA20–CB40 and reference sample.

Cut resistance measures how the specimen resists the growth of cuts. The cut resistance study was carried out using pre-cut samples. The strength at the break of pre-cut samples and cut sizes was recorded. According to Griffith [30,31], the fracture energy *G_c_* follows the expression

(3) $$\sigma_{b}={\left(G_{c}E/\pi l\right)}^{1/2}$$

where $\sigma_{b}$ is the stress at break, Gc is the fracture energy, E is Young’s modulus and *l* is the cut size. Thus, the tensile strength at break for each pre-cut specimen was plotted against the cut size on a double logrithmic scale. The double-log plot directly compared the fracture energy of vulcanizate with the same Young’s modulus, *E*. As shown in Figure 5, βA20–CB40 followed the Griffith fracture theory. In the log–log plot, the strength decreased steadily as the cut size increased. The results showed that βA20–CB40 was appreciably more resistant to cuts than the reference sample. Though the normal tensile strength of βA20–CB40 and the reference sample were presumably the same, the breaking stress of βA20–CB40 with a larger cut size was obviously higher than that of the reference sample.

### 3.4. Dynamic Mechanical Analysis

**Figure 6:**
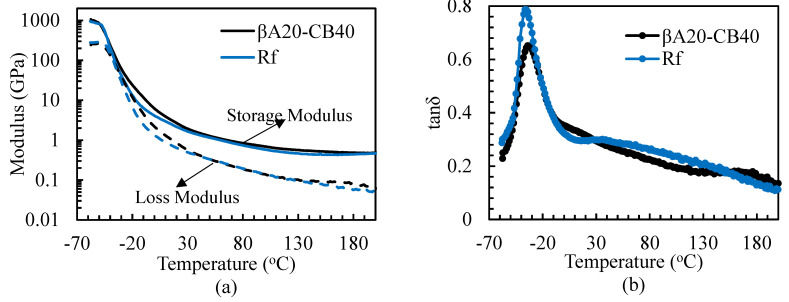
Dynamic mechanical analysis of βA20–CB40 and reference sample. The tests were carried out in a tension mode at 1 Hz and 2% strain. (**a**) Storage and loss modulus as a function of temperature. (**b**) Loss factor as a function of temperature.

The dynamic mechanical analyses of βA20–CB40 and the reference sample were carried out in a tension mode at 1 Hz and 2% strain. Their storage modulus (E’), loss modulus (E”) and loss factors (tanδ) as a function of temperature are shown in Figure 6. Since tanδ levels at 0 °C and 60 °C are commonly taken as indicators for the wet skid resistance and rolling resistance of tire tread, they are compiled in Table 5. As shown in Table 5, βA20–CB40 showed significantly lower rolling resistance (11%) than the reference sample with higher wet skid resistance (9%), indicating that the use of the supramolecular filler was able to improve fuel efficiency without sacrificing wet-skid resistance. As shown in Figure 6b, the tan δ peak at around 180 °C for βA20–CB40 coincided with the phase transition detected by DSC, indicating the melting of A_3_ crystals. The area under the tan δ peak for βA20–CB40 at the glass transition area was lower than that of the reference sample, as expected, since the volume fraction of polymer chains for the two samples follows the same trend and hysteresis in the glass transition zone is mainly attributed to the molecular friction between chains.

**Figure 7:**
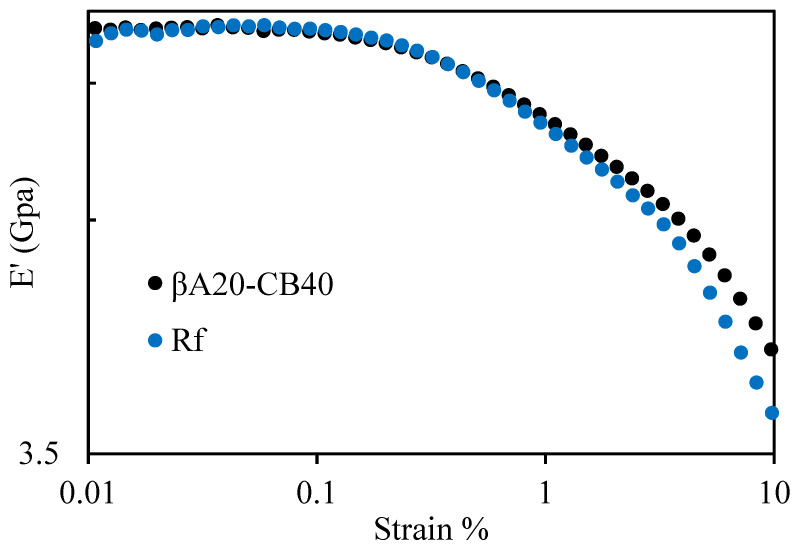
Strain dependance of storage modulus for βA20–CB40 and reference sample. The test was carried out in tension mode at 1 Hz, and the strain amplitude was from 0.01% to 10%.

The Payne effect was characterized by a strain sweep test, and the test was carried out at a frequency of 1 Hz at room temperature (25 °C). The strain amplitude was from 0.01% to 10%. The Payne effect was mainly attributable to the disruption of the filler–filler interaction for filler-reinforced elastomers [32,33,34]. The Payne effect is practically undesirable because it lowers fuel efficiency while not contributing to durability [23,35]. As shown in Figure 7, the Payne effect of βA20–CB40 was less pronounced than that of the reference sample. The result can be explained as follows; the oligo(β-alanine) supramolecular domains in βA20–CB40 were expected to compensate for the breakage of the filler–filler interaction of carbon black and, as a result, delayed the reduction of the storage modulus and reduced the Payne effect.

### 3.5. Effect of Mixing Method

The crosslink densities of vulcanizates obtained from the swelling test are shown in Table 6. The crosslink density of βA20–CB40HT was slightly higher than that of βA20–CB40. This phenomenon was presumably because of the different mixing temperature. The proton on the carbon that is next to sulfur is active, and as a result the A_3_-SBR pristine polymer can more easily have a crosslink reaction than the unfunctionalized SBR. Therefore, βA20–CB40HT with a high mixing temperature was partially crosslinked during mixing.

**Figure 8:**
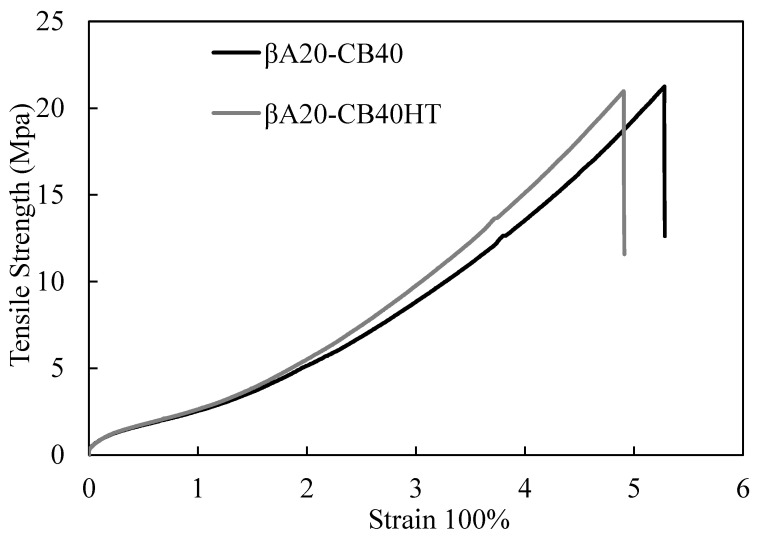
Comparison of tensile properties between βA20–CB40 and βA20–CB40HT.

Mixing methods result in differences in mechanical properties, as shown in Figure 8. βA20–CB40HT mixed at a high temperature showed a lower extension and higher modulus, which could be explained as follows: partial crosslinking happens during the high temperature rubber compounding process, and this results in a higher crosslink density of βA20–CB40HT vulcanizates. Therefore, the strain hardening of βA20–CB40HT happens earlier than that of βA20–CB40. As a result, βA20–CB40HT shows higher modulus at 100% strain and 300% strain.

## 4. Conclusions

Oligo(β-alanine)s supramolecular fillers were successfully mixed with the SBR matrix by synthesizing the β-alanine trimer-grafted SBR (A_3_-SBR) pristine polymer and mixing the pristine polymer with the SBR matrix to help dispersion. The pristine polymer (A_3_-SBR) needed to be saturated with ethanol and mixed at a low temperature to obtain good miscibility and prevent crosslinking during rubber compounding. A Differential Scanning Calorimetry study showed that the melting temperature of β-sheet crystals in βA20–CB40 was around 179 °C and was broad. The melting temperature was similar to that of the pristine polymer, and the broad melting peak likely suggests that the size of the crystals was not uniform. The Transmission Electron Microscopy study revealed that in βA20, some β-sheet crystals are rod-like with several tens of nanometers and some β-sheet crystals are particulate with low aspect ratios. A mechanical study and dynamic mechanical analysis showed that vulcanizates containing A_3_-SBR were significantly less hysteretic at 60 °C, more hysteretic at 0 °C based on Tan δ, and more cut-resistant based on tensile testing with pre-cut specimens. Overall, the “magic triangle” was expanded by optimizing the rolling resistance, wet-skid and cut resistance simultaneously using the oligo(β-alanine)s supramolecular filler. The Payne effect became less pronounced after introducing the oligo(β-alanine)s supramolecular filler.

## Figures and Tables

**Table 1:** Formulation of rubber compounds (unit: phr).

Name	βA20–CB40/βA20–CB40–HT	Rf	βA20	Notes
Styrene butadiene copolymer (SLF16S42)	50	70	50	Matrix
PBD	30	30	30	
A_3_-SBR	20	---	20	
CB-N115	40	40	---	Filler
PD-2	1.5	1.5	1.5	Antioxidant
Antioxidant DQ	1	1	1	
Stearic acid	1.8	1.8	1.8	Activator
Zinc oxide	3.5	3.5	3.5	
Akrowax (Micro23)	1	1	1	Plasticizer
Retarder CTP	0.1	0.1	0.1	Retarder
Sulfur	1.75	1.93	1.75	Curing agent
BBTS	0.75	0.83	0.75	Accelerator

**Table 2:** Curing characteristics of βA20–CB40, βA20–CB40–HT, βA20 and Rf.

Sample Name	M_L_ (dNm)	M_H_ (dNm)	t_s2_ (min)	t_90_ (min)	Cure Rate Index * (min^−1^)
βA20–CB40	2.46	16.93	4.56	12.5	12.6
βA20–CB40–HT	2.65	17.14	3.83	12.02	12.2
βA20	1.55	9.41	6.22	13.4	13.9
Rf	2.19	16.87	5.92	15.76	10.2

* Cure rate index = 100/(t_90_ − t_s2_).

**Table 3:** Elemental analysis result for A_3_-SBR pristine polymer.

Sample Name	C (wt %)	H (wt %)	N (wt %)	S (wt %)
A_3_-SBR	84.52	10.68	1.20	0.60

**Table 4:** Summary of tensile properties of βA20–CB40 and reference sample.

Name	δ_100%_ (MPa)	δ_300%_ (MPa)	δ_b_ (MPa)	ε_b_ (%)	Toughness (J/cm^3^)
βA20–CB40	2.55 ± 0.09	8.83 ± 0.05	21.25 ± 1.1	527 ± 8	45 ± 2
Rf	2.21 ± 0.08	8.79 ± 0.10	21.16 ± 1.2	526 ± 10	45 ± 2

The data are the average of three tests. Numbers in the parentheses are the range of experimental error.

**Table 5:** Summary of loss factor at 0 °C and 60 °C for βA20–CB40 and reference sample.

	βA20–CB40	Rf
Tan δ at 0 °C	0.354	0.325
Tan δ at 60 °C	0.251	0.282

**Table 6:** Swelling test results for βA20–CB40 and βA20–CB40HT.

	Crosslink Density mol/m^3^	Molecular Weight between Crosslinks g/mol
βA20–CB40	146	6100
βA20–CB40HT	151	6300

## Data Availability

Not applicable.
